# HIV-1 capsid is the key orchestrator of early viral replication

**DOI:** 10.1371/journal.ppat.1010109

**Published:** 2021-12-30

**Authors:** Vojtech Zila, Thorsten G. Müller, Barbara Müller, Hans-Georg Kräusslich

**Affiliations:** 1 Department of Infectious Diseases, Virology, Heidelberg University, Heidelberg, Germany; 2 German Center for Infection Research, Partner Site Heidelberg, Heidelberg, Germany; Medical Research Council Laboratory of Molecular Biology, UNITED KINGDOM

## Introduction

**Fig 1:**
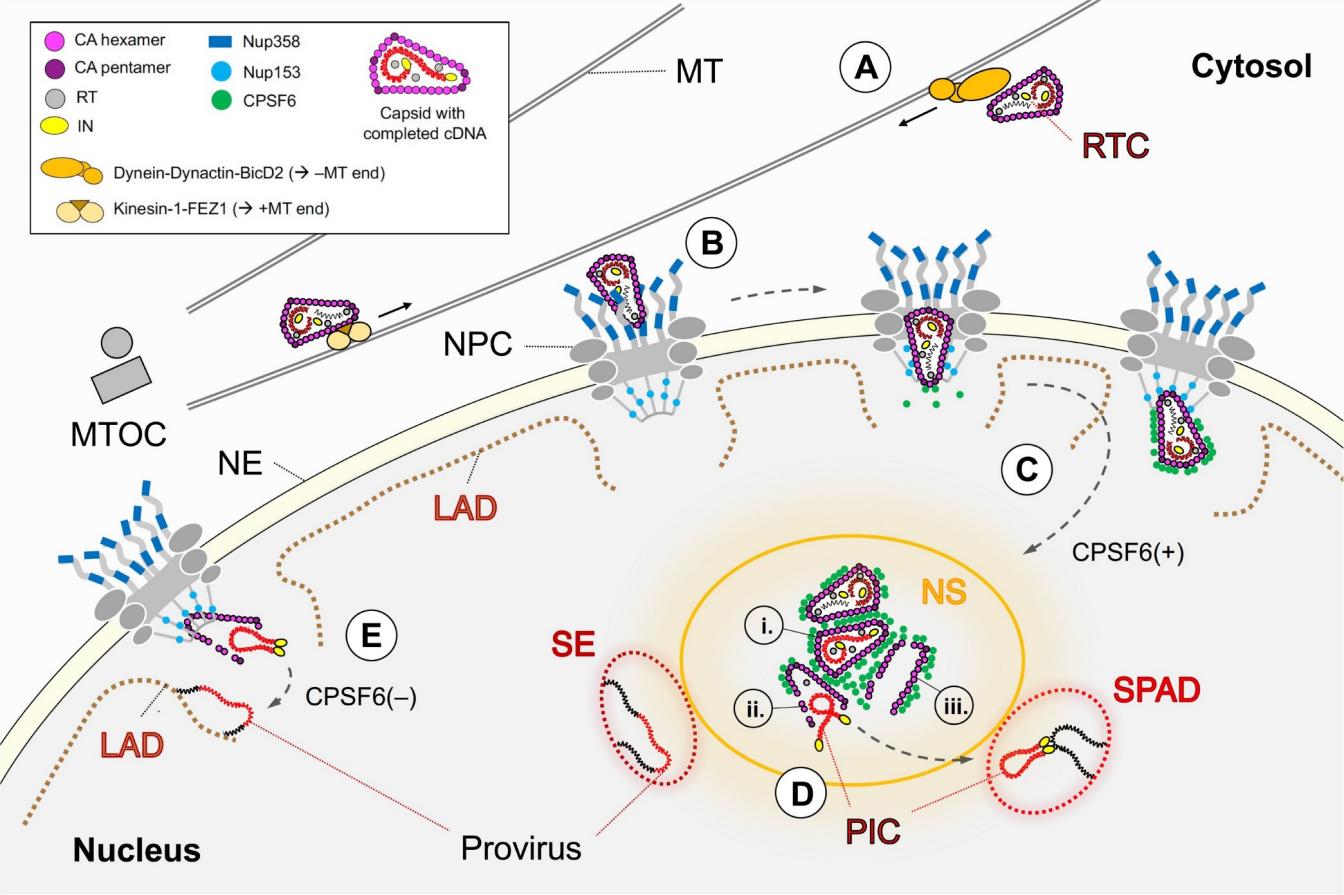
Early postentry steps of HIV-1 replication. **(A)** After fusion-mediated release of the HIV-1 capsid into the cytosol, reverse transcription of the viral RNA genome into double-stranded cDNA commences (RTC), and the capsid interacts with dynein and kinesin-1 motor complexes for transport along MTs toward the nuclear envelope (reviewed in [14]). **(B)** At the cytosolic side of NPCs, MT-associated capsids encounter Nup358 (blue rectangles), a major component of filaments emanating from the NPC. After docking at the nuclear pore with their narrow end, capsids then penetrate into the NPC central channel, which is sufficiently dilated to accommodate the complete capsid. **(C)** Once exposed to the nucleoplasm, sequential interaction of the CA lattice with Nup153 (blue dots) and CPSF6 (green dots) (and possibly other factors) releases the capsid into the nucleoplasm. CPSF6 mediates accumulation of capsids in NSs, where cDNA synthesis is completed (i). Inside the nucleus, the capsid structure is physically disrupted and the PIC is released (ii). **(D)** The PIC then separates from the capsid remnants (iii) and integrates into the host cell genome in SPADs or SE domains (reviewed in [10,31]). **(E)** In the absence of CPSF6, the capsid remains close to the nuclear basket and does not efficiently enter the nucleoplasm. Completion of cDNA synthesis and uncoating occur at this site, followed by breakage of the capsid and genome integration into LADs [10]. CA, capsid protein; HIV-1, human immunodeficiency virus 1; LAD, lamin-associated domain; MT, microtubule; MTOC, microtubule organizing center; NE, nuclear envelope; NPC, nuclear pore complex; NS, nuclear speckle; PIC, preintegration complex; RTC, reverse transcription complex; SE, super-enhancer-rich domain; SPAD, speckle-associated domain.

The cone-shaped capsid enclosing the viral RNA genome and the replication machinery is the hallmark of mature human immunodeficiency virus 1 (HIV-1). Over the past decade, our understanding of capsid function in the early events following membrane fusion and cytosolic entry underwent a major paradigm shift. In particular, recent studies using microscopic approaches shed new light on the involvement of the capsid structure in postentry replication and resolved some apparent discrepancies raised by earlier reports. The emerging model of the early phase of HIV-1 replication places the capsid in the leading role as key orchestrator of viral replication steps and HIV-1–cell interactions during the postentry phase. Here, we summarize how recent observations have reshaped our view on HIV-1 postentry (Fig 1).

## Discard after entry? The traditional picture of the uncoating process

The most typical feature of mature, infectious HIV-1 particles is the emblematic conical capsid. This structure consists of a homomultimer of the viral CA (capsid) protein, which assembles into a closed fullerene structure consisting of approximately 250 CA hexamers and 12 CA pentamers, 7 at the wide and 5 at the narrow end of the cone (reviewed in [1,2]). This elaborate structure is not initially formed during virus assembly: Particle formation is directed by the viral Gag polyprotein consisting of multiple folded domains, including CA. Upon release of the immature, noninfectious particle, Gag is cleaved at multiple sites by the viral protease, yielding fully processed CA, which subsequently assembles into the mature CA lattice inside the complete virion (reviewed in [1]). Accordingly, processed CA and the mature CA lattice are not needed for virus assembly or release, pointing toward a function of the mature lattice structure during or after virus entry. This hypothesis was supported by several early reports that identified mutations in the CA region associated with defects exclusively in the early phase of HIV-1 replication or mapped the effects of HIV-1 restriction factors to the viral CA protein and CA lattice (reviewed in [1]).

Proteolytic maturation is traditionally understood as a solution for the assembly–disassembly paradox [3]: Stable virions assembled in the producer cell are converted into a metastable state, ready to rapidly release the viral genome upon cell entry. Accordingly, early schemes of HIV-1 replication assumed rapid and complete disintegration (uncoating) of the CA lattice after membrane fusion with subsequent cytoplasmic conversion of the genomic RNA into double-stranded cDNA in the released reverse transcription complex (RTC) (reviewed in [4]). The resulting preintegration complex (PIC) was suggested to enter the nucleus through intact nuclear pores, but the relevant nuclear import factors remained enigmatic. Rapid uncoating after membrane fusion appeared to be supported by the rapid and complete dissociation of the capsid when the membrane of cell-free virions was stripped by detergent and by the failure to detect cone-shaped objects in the cytosol of infected cells by electron microscopy (EM) (reviewed in [5]). Moreover, early replication complexes purified from extracts of infected cells contained little or no CA [5]. Immediate postentry uncoating could not explain, however, why the elaborate structure of the fullerene capsid had evolved, although it appeared to be dispensable for both virus morphogenesis and the early replication phase. Furthermore, mutational and microscopic analyses provided evidence for a functional and temporal link between capsid uncoating and reverse transcription, suggesting a more gradual uncoating process (reviewed in [5]). Recent advancements in fluorescence and EM based imaging approaches in conjunction with novel labeling strategies and elegant in vitro approaches helped to resolve the conundrum presented by apparent discrepancies between earlier studies.

## A fresh look: New insights from biochemical and imaging studies

One main argument for rapid uncoating was the instability of the mature capsid observed in biochemical analyses. An important advance in our understanding came from the observation that the HIV-1 capsid is actually a stable structure in the presence of inositol hexakisphosphate (IP6) [6,7]. IP6 binds to and coordinates basic side chains in a pore on the surface of the mature CA lattice; this increases the half-life of the assembled capsid after detergent removal of the viral membrane from minutes to many hours. Adding deoxynucleoside triphosphates (dNTPs) to this stabilized structure in vitro even allowed complete reverse transcription of the endogenous viral genome and revealed breakage, but not complete disassembly of the lattice, when synthesis of the double-stranded cDNA approached completion [8]. Since IP6 is present in high concentration in the cytosol, these in vitro data clearly indicate that the capsid structure could remain intact for prolonged periods of time after membrane fusion and could support the full process of reverse transcription once dNTPs become available. But does this also occur in infected cells?

Various methods have been used to identify and track viral replication complexes and their association with CA in different subcellular compartments (reviewed in [9]). They include immunofluorescence using different antibodies, incorporation of fluorescent fluid phase markers or fluorescently tagged proteins of the replication complex into the virion, as well as labeling of newly synthesized viral cDNA, indirect labeling of the capsid, and incorporation of substoichiometric amounts of CA–GFP fusion proteins into the capsid. Consistently, these studies confirmed that the incoming capsid stays intact and remains associated with the viral replication complex at least for some time after membrane fusion. Conclusions regarding time and location of eventual uncoating were highly divergent, however. Some studies reported delayed cytoplasmic uncoating with residual CA molecules retained on the RTC, while other groups suggested uncoating close to or directly at the nuclear pore, and yet other groups provided evidence for nuclear import of largely intact capsids and nuclear uncoating (reviewed in [2,9,10]). Importantly, these conclusions were generally based on indirect detection methods, which may explain some of the observed differences, albeit the possibility of multisite uncoating cannot be excluded at present.

EM or electron tomography (ET) analysis could directly identify cytosolic structures, but previous studies yielded at best anecdotal evidence for conical structures in the cytosol of infected cells. This is not surprising, however: Detection of rare subviral structures against the dense background of the surrounding cytosol is comparable to finding a needle in a haystack. Three-dimensional correlative light and electron microscopy (CLEM), by which objects of interest are first localized using fluorescence imaging and then characterized by ET, can overcome this problem. Recent advances provided a powerful tool to analyze the three-dimensional architecture of complex biological objects directly in their cellular context. Employing this approach, we have identified numerous apparently intact postfusion HIV-1 capsids in the cytoplasm and in close vicinity to the nuclear envelope; almost all fluorescently labeled cytoplasmic HIV-1 RTC could be correlated with a typical capsid cone by CLEM [11]. This result is consistent with other studies reporting strong CA signals on cytoplasmic RTC resembling the signal of the complete virion core (reviewed in [9]). We thus hypothesize that the HIV-1 capsid stays intact—or at least largely intact—during cytoplasmic trafficking, and enclosure of the RTC inside the capsid protects the HIV-1 genome, forms a reaction container for reverse transcription, and shields the nascent cDNA from innate sensing. Consistent with this model, induction of premature uncoating in the cytosol induced innate immune signaling and led to proteasomal degradation of subviral complexes [12,13].

## Finding the key in the pocket: Interactions between the capsid and the nuclear pore

Following membrane fusion, apparently intact HIV-1 capsids travel along microtubules (MTs) toward the nuclear envelope. Trafficking requires dynein and kinesin-1 motors, which interact with the CA lattice through motor adaptor proteins (reviewed in [10,14]). Once at the nuclear periphery, kinesin-1 has been reported to facilitate interaction of the capsid with the nucleoporin Nup358 (RanBP2), relocated from the nuclear pore complex (NPC) to the cytosol. The latter interaction involves the cyclophilin domain of Nup358 and the flexible cyclophilin binding loop of CA that is exposed on the surface of the capsid (reviewed in [10,14]). A direct role of the microtubular network until capsid arrival at the nuclear envelope is consistent with our CLEM analyses: Most conical capsids in the vicinity of the nuclear envelope were closely associated with MTs; capsids were frequently found in multiples, suggesting that they arrived via common MT transport routes [11].

Once at the nuclear envelope, HIV-1 capsids appear to directly interact with the cytoplasmic face of nuclear pores, probably mediated by interaction with the cyclophilin domain of Nup358. Interestingly, tomogram renderings of capsids docked at the NPC revealed that the narrow end of the cone was almost always positioned toward the central NPC channel [11]. It is tempting to speculate that this orientation may be governed by the higher density of CA pentamers at the narrow end of the fullerene cone. Preferential interaction of highly curved capsid regions with Nup358 has been suggested recently [15], but other host factors may also be involved. Preferential interaction of the narrow end with the NPC may guide the orientation of the capsid, thereby initiating the nuclear import process—threading the narrow tip of the cone into the NPC channel could facilitate entry into the narrow gate. We hypothesize that this mechanism may also provide some explanation for the distinct conical shape of the HIV-1 capsid.

## Stop right here: What happens at the nuclear pore?

Besides Nup358, HIV-1 CA has been shown to also interact with other nucleoporins, including Nup153, Nup214, Nup88, Nup62, Nup98, and Nup107 (reviewed in [10]). Nup153, an inner nuclear basket protein, binds to a hydrophobic pocket in the assembled CA lattice, and binding is suggested to be mediated by the Phe–Gly (FG) repeats of the nucleoporin. FG repeats are common to several nucleoporins and form a disordered gel-like structure that fills the central channel of the NPC and conducts all nuclear import processes (reviewed in [16]). Conceivably, the HIV-1 capsid may thus also interact with FG repeats of other nucleoporins besides Nup153. Since Nup153 is a nuclear basket component located at the nuclear side of the NPC, its interaction with the CA lattice would suggest that at least a partially assembled lattice—if not the complete capsid—is retained throughout nuclear import.

The main argument against nuclear import of the complete HIV-1 capsid has been its size: With a width of approximately 60 nm at the wide end, the CA appeared to be too large for translocation through the central NPC channel with a reported diameter of approximately 40 nm (reviewed in [2]). A second argument was the variable detection of CA on nuclear subviral HIV-1 complexes, which yielded low or undetectable signals in most studies (reviewed in [5,9]). Therefore, it seemed clear that uncoating is a prerequisite for passage of the subviral complex through the NPC. This conclusion was challenged by immunofluorescence analyses, which revealed strong CA signals on nuclear subviral HIV-1 complexes in macrophages [17]. CA signals were initially not detected in nuclei of infected T cells and variably detected in other cell-types, but this can now be explained by different accessibility of nuclear and cytoplasmic CA structures to antibody detection. Nuclear structures are highly decorated with large clusters of the cellular protein cleavage and polyadenylation specificity factor 6 (CPSF6; see below) and possibly other host cell proteins, which mask CA epitopes and thus prevent detection of the underlying capsid structure. This can be overcome by treatment with the small molecule PF74 (competing for CPSF6 binding to the CA lattice) or by different extraction treatments [18]. These data, as well as fluorescent labeling of CA fusion proteins [19], indicated that postfusion cytoplasmic and nuclear HIV-1 subviral structures contain comparable amounts of CA.

**Fig 2:**
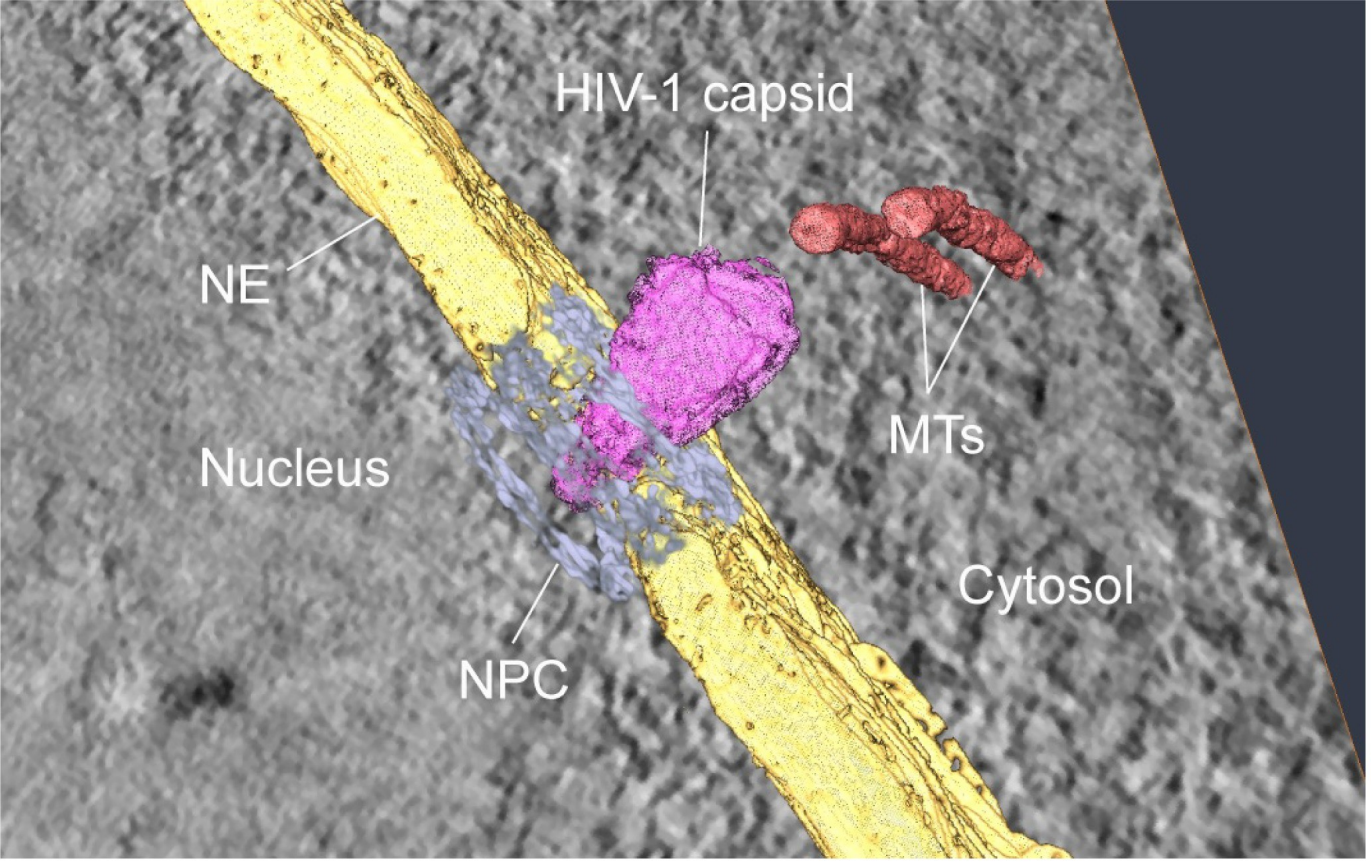
Nuclear import of HIV-1 CA visualized by ET. Three-dimensional rendering of electron tomographic reconstruction shows the HIV-1 capsid penetrating the NPC central channel in an infected T lymphoblast. ET, electron tomography; HIV-1, human immunodeficiency virus (1); NE, nuclear envelope; NPC, nuclear pore complex; MT, microtubule. *Adapted from Lucic and colleagues [31]*.

The described results leave the options that either the HIV-1 capsid is remodeled to fit through the NPC, the NPC may widen for capsid transport, or both. Recent CLEM and cryo-ET analyses resolved these questions, at least in part. Cryo-ET of nuclear pores in uninfected and infected T cells revealed that the diameter of the NPC central channel in situ is larger (approximately 64 nm) than reported in previous analyses of isolated nuclear pores [11]. It is thus sufficiently wide to accommodate the entire HIV-1 capsid. Apparently intact cone-shaped capsids containing dense nucleoprotein complexes were observed inside the NPC central channel (always with the narrow end pointing toward the nucleus; Fig 2) [11]. CLEM and cryo-ET analyses of nuclear subviral complexes rarely revealed cone-shaped structures enclosing dense nucleoprotein complexes, however. Most structures identified by correlative analyses appeared to be empty inside, sometimes retaining a cone shape, but often exhibiting tubular or irregular morphologies [11]. These data indicate that apparently complete HIV-1 capsids enter and pass through the NPC channel, but do not allow firm conclusions regarding alteration of the capsid during this passage.

The Nup153 binding pocket on the CA is also the target of the nuclear host protein CPSF6, which has been reported to be a dependency factor promoting HIV-1 replication (reviewed in [10]). Super-resolution microscopy revealed recruitment of CPSF6 to HIV-1 cDNA containing complexes at the nuclear side of the NPC [17] and knockdown of CPSF6 or a mutation in the CPSF6 binding site of CA arrested subviral complexes at the nuclear pore [17]. Interestingly, CPSF6 is not essential for nuclear translocation or infectivity, and subviral complexes in the absence of CPSF6 binding accumulated directly adjacent to nuclear pores accompanied by viral genome integration into lamina-associated chromatin (reviewed in [10]). The same site in the CA lattice has recently also been reported to interact with the cytosolic host protein Sec24C [20]. Based on these observations, we hypothesize that there may be a handoff of HIV-1 capsids as they traffic toward the cell nucleus with consecutive (and potentially competitive) binding of several host factors (Sec24C–Nup153–CPSF6) to a highly reactive pocket in the CA lattice.

Based on the described results, we suggest a model for nuclear entry of the HIV-1 capsid: Following MT-mediated transport, the pentamer-rich narrow end of the capsid directly associates with Nup358 at the cytoplasmic face of a nuclear pore. Positioned into the central channel, the array of FG repeat binding pockets on the capsid surface mediates repetitive interactions with FG repeat nucleoporins throughout the central channel. The number of binding sites increases toward the wide end of the cone, leading to immersion of the capsid into the central NPC channel, with multivalent, low-affinity binding to FG repeats within the channel driving the capsid toward the nuclear side. Once the narrow tip reaches the nuclear basket, CPSF6 binding competes for and obscures the FG binding pocket on the CA lattice. CPSF6 binding may thus provide a ratchet type mechanism aiding release from the NPC. The latter is not essential for genome delivery into the nucleus, but may be kinetically relevant and also appears to be important for subsequent nucleoplasmic trafficking (see below). In this model, the entire HIV-1 capsid acts as an unconventional multivalent nuclear import machinery driving nuclear entry of large subviral cargo without requiring conventional nuclear import factors.

## It’s time to move on: Separation of the viral genome from the capsid in the nucleus

Following nuclear import, CPSF6 clustering on the CA lattice of subviral complexes has been reported to mediate nucleoplasmic trafficking to nuclear speckle domains [21–23]. Multiple replication complexes accumulate at these sites, and CLEM analysis also revealed small clusters of closely apposed nuclear capsid-like structures, both conical and tubular in appearance, upon high multiplicity infection [18]. It appears likely, therefore, that the entire capsid or capsid-like structure containing the (partially) reverse transcribed genome and replication machinery travels via CPSF6 to distinct subnuclear sites, where integration occurs. For chromatin integration, the capsid needs to open, and the cDNA with associated integrase (and potentially other factors) has to be released.

Nuclear uncoating has been studied by tracking of fluorescently labeled HIV-1 cores in living cells and by labeling the newly synthesized HIV-1 cDNA as well as components of the replication machinery. The former study indicated that uncoating occurs rapidly in close vicinity to the subsequent integration site, <1.5 hours before integration [19]. Fluorescent 2-color imaging and CLEM of HIV-1 dsDNA and a component of the replication machinery confirmed clustering in selected nuclear locations and revealed separation of the genomic viral DNA complex from the bulk of associated proteins (including most, if not all, of CA and CPSF6) over time [18]. Broken capsid-related structures were observed by CLEM in the position of the viral proteins after this separation, while the exposed viral cDNA appeared as dense elongated structure, morphologically resembling chromatinized DNA [18]. Based on these results, we hypothesize that the capsid does not cooperatively disassemble as previously suggested, but appears to break open once it reaches its final destination in nuclear speckles. The discrepancy to the labeling studies, which suggested loss of the CA lattice [19,24], might be explained by the fact that the latter made use of a fluorescent CA fusion protein, which was incorporated in substoichiometric amounts and may be preferentially lost upon capsid disintegration. Further studies will be required to fully resolve this apparent discrepancy.

Based on these data, we can conclude that the capsid with associated proteins eventually breaks open inside the nucleus and releases the viral genome and integration factors. This appears to occur in close vicinity of the actual integration site and does not appear to require cooperative disassembly of the CA lattice. What then triggers breakage of the capsid? One option is completion of reverse transcription. Multiple recent studies indicated that viral cDNA synthesis is completed inside the nucleus [18,19,21–23,25]. dsDNA has different mechanical properties and requires a larger volume than the template ssRNA strands. Reverse transcription of the viral genome may thus lead to breakage of the capsid, once DNA synthesis reaches completion. Mechanical strain from the growing dsDNA as a trigger or driver of the uncoating process has been proposed in several earlier studies (reviewed in [26]) and is consistent with the detection of a partially broken CA lattice with emanating nucleic acid upon endogenous reverse transcription in isolated capsids in vitro [8]. It should be noted, however, that HIV-1-based vectors with much shorter genomes effectively transduce nondividing cells. Furthermore, the HIV-1 NC (nucleocapsid) protein has been shown to compact DNA [27] and may thus help to overcome the strain imposed by synthesis of the more rigid cDNA. Further studies will also be needed to determine whether and how the CA lattice may be (partially) destabilized during passage through the narrow nuclear pore channel and whether this could facilitate its subsequent breakage. Obviously, additional host cell factors at the final destination of nuclear speckles may also be involved. Genomic HIV-1 cDNA is rapidly chromatinized when becoming accessible inside the nucleus [28], and this is consistent with morphological appearance of released genomes by CLEM [18]. It is tempting to speculate, therefore, that immediate chromatinization of the viral cDNA, once it becomes accessible from inside the broken capsid, could provide a driving element for complete uncoating of the viral replication complex.

## Conclusions

In summary, it has become clear that the HIV-1 capsid represents much more than a delivery container for the viral genome. It provides a closed environment for reverse transcription, protects the nascent viral cDNA from DNA sensors, and acts as delivery vehicle mediating transport toward and through the nuclear pore and even within the nucleus. These key functions render the mature capsid a promising target for antiviral drugs. Several capsid binding small molecules have already been developed into promising drug candidates, with the highly potent long-acting capsid inhibitor GS-6207 (lenacapavir) [29,30] in Phase II/III clinical trials (reviewed in [2]).
